# Differential Sensitivity of Endocrine and Non-Endocrine Tissues to Cadmium-Induced Lipid Peroxidation and the Protective Role of Melatonin †

**DOI:** 10.3390/ijms27135991

**Published:** 2026-07-03

**Authors:** Aleksandra K. Gładysz, Jan Stępniak, Małgorzata Karbownik-Lewińska

**Affiliations:** 1 Department of Endocrinology and Metabolic Diseases, Medical University of Lodz, 281/289 Rzgowska St., 93-338 Lodz, Poland; aleksandra.gladysz@umed.lodz.pl (A.K.G.); jan.stepniak@umed.lodz.pl (J.S.); 2 Polish Mother’s Memorial Hospital—Research Institute, 281/289 Rzgowska St., 93-338 Lodz, Poland

**Keywords:** melatonin, cadmium, oxidative damage, thyroid, antioxidant, carcinogen, endocrine-disrupting chemical

## Abstract

Cadmium is a toxic heavy metal classified by the International Agency for Research on Cancer as a human carcinogen and recognized as an endocrine-disrupting chemical. The present study aimed to evaluate tissue-specific susceptibility to cadmium-induced oxidative damage to membrane lipids (lipid peroxidation, LPO) and to assess the antioxidative effects of melatonin in porcine tissue homogenates representing endocrine (the thyroid and the ovary) and non-endocrine (the liver, the kidney, and the brain) organs. Homogenates were incubated with cadmium chloride (CdCl_2_; 2.5–1000 µM) without/with melatonin (0.1–5.0 mM). Lipid peroxidation was assessed spectrophotometrically by measuring malondialdehyde + 4-hydroxyalkenals (MDA + 4-HDA) levels. Cadmium significantly increased LPO in the liver (2.5–1000 μM) and in the kidney (25–1000 μM), whereas no prooxidative effect was observed in endocrine tissues or in the brain. Liver damage was mitigated by melatonin doses as low as 0.1 μM across the 250–1000 μM cadmium range, while protection in the kidney was limited to higher melatonin concentrations (2.5–5.0 mM) against damage induced by 100–1000 μM cadmium concentrations. The findings demonstrate pronounced tissue-specific differences in susceptibility to cadmium-induced oxidative stress and support the potential of melatonin as a preventive agent against heavy metal-induced oxidative stress, particularly in non-endocrine organs.

## 1. Introduction

Exposure to heavy metals is recognized as a major environmental concern with significant implications for public health [1]. These elements are released into the environment primarily as a result of industrial activities, including mining, smelting, and fossil fuel combustion [2], as well as intensive agricultural practices and ongoing urbanization [3]. Consequently, heavy metals can accumulate in environmental compartments such as air, soil, and water, from which they enter the human body mainly through inhalation and dietary intake [4,5]. Due to their ability to bioaccumulate in tissues and organs, long-term exposure may disrupt physiological processes and contribute to the development of chronic toxic effects [6].

The International Agency for Research on Cancer (IARC) has classified cadmium (Cd) among substances recognized as carcinogenic to humans (Group 1) [7]. Prolonged exposure to this heavy metal is associated with the induction of oxidative stress and inflammatory responses in the body. Cadmium is also recognized as an endocrine-disrupting chemical (EDC), as it can mimic, block, or modulate the activity of endogenous hormones, thereby disrupting normal endocrine signaling pathways and hormonal homeostasis [8,9].

Following absorption, cadmium exerts toxic effects on multiple organs and systems, with particular vulnerability observed in tissues involved in metabolism and detoxification processes. Owing to its long biological half-life and pronounced capacity for tissue accumulation, cadmium may persist in the body for decades, and its toxic effects can be sustained over time, contributing to an increased risk of chronic metabolic and endocrine disorders [10,11].

Given the central role of oxidative stress in cadmium-induced toxicity, increasing attention has been paid to the potential protective effects of antioxidants that can counteract free radical-mediated damage. Among them, melatonin (N-acetyl-5-methoxytryptamine) has emerged as a particularly promising candidate. It is a widely distributed molecule, synthesized by a broad range of organisms, and is best known for its role in the regulation of circadian rhythms [12,13]. In addition, melatonin exhibits strong antioxidant properties, acting both as a direct free radical scavenger [14] and as a modulator of antioxidant defense systems, thereby protecting cellular macromolecules under various experimental conditions [15,16,17,18,19,20].

Although cadmium-induced oxidative stress has been extensively documented, considerably less is known about tissue-specific differences in susceptibility to cadmium-induced lipid peroxidation, particularly when endocrine and non-endocrine organs are compared under identical experimental conditions. Endocrine tissues differ substantially from non-endocrine organs with respect to their physiological redox characteristics, metabolic activity, and susceptibility to oxidative damage. Therefore, comparative evaluation of cadmium-induced oxidative stress in different tissues may contribute to a better understanding of organ-specific vulnerability to this environmental toxicant. Furthermore, despite numerous reports demonstrating the antioxidative properties of melatonin [14,15,16,17,18,19,20], its protective efficacy against cadmium-induced lipid peroxidation has not been systematically compared across endocrine and non-endocrine tissues in a single experimental model.

The thyroid and the ovary were selected as representative endocrine organs because they differ in their physiological redox characteristics. In the thyroid gland, physiological oxidative processes are indispensable for thyroid hormone synthesis [21], whereas the ovary is not characterized by such intensive constitutive oxidative processes related to hormone synthesis [19]. Additionally, both are recognized targets of cadmium-induced endocrine disruption. The liver and the kidney were included as major sites of cadmium accumulation and toxicity, whereas the brain was selected due to its well-known susceptibility to oxidative stress. Therefore, the selected tissues represent organs with distinct physiological functions and different potential vulnerability to cadmium-induced oxidative damage.

The study aimed to check tissue sensitivity to cadmium used in the form of cadmium chloride (CdCl_2_) and to assess whether, and to what extent, melatonin can prevent cadmium-induced oxidative damage to membrane lipids (lipid peroxidation, LPO). This investigation was conducted using porcine tissue homogenates from two endocrine organs, i.e., the thyroid and the ovary, as well as three non-endocrine organs, i.e., the liver, the kidney, and the brain.

## 2. Results

**Figure 1:**
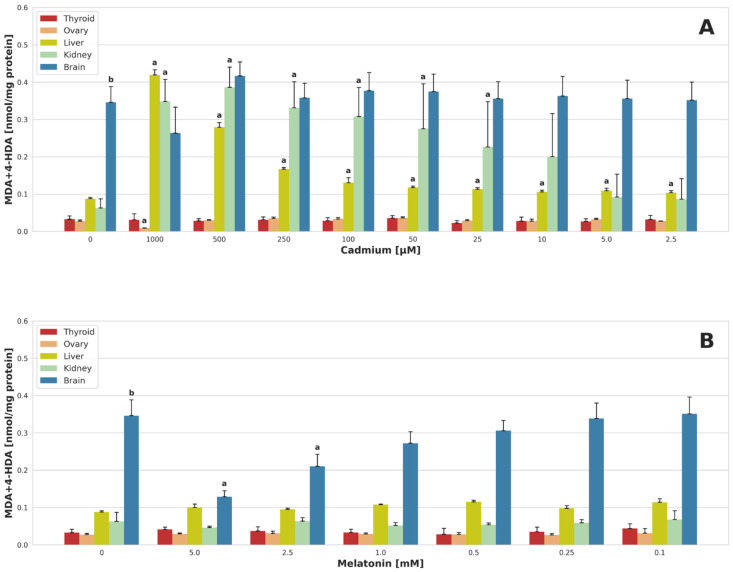
Lipid peroxidation (LPO), assessed as the level of malondialdehyde + 4-hydroxyalkenals (MDA + 4-HDA), in porcine tissue homogenates. Homogenates were incubated in the presence of (**A**) cadmium chloride (CdCl_2_) (1000, 500, 250, 100, 50, 25, 10, 5.0 and 2.5 µM); (**B**) melatonin (5.0, 2.5, 1.0, 0.50, 0.25 and 0.10 mM). LPO levels are expressed as nmol/mg protein. Data are presented as mean ± SE (error bars). “a” *p* < 0.05 vs. control within the same tissue; “b” *p* < 0.05 vs. control values in all other tissues.

Incubation of porcine tissue homogenates with CdCl_2_ alone resulted in tissue-specific changes in LPO, measured as malondialdehyde + 4-hydroxyalkenals (MDA + 4-HDA levels) (Figure 1A). A concentration-dependent increase in LPO was observed predominantly in liver and kidney homogenates, reaching statistical significance across the entire range of tested CdCl_2_ concentrations (1000–2.5 µM) in the liver and within the range of 1000–25 µM in the kidney, whereas the thyroid, the ovary, and the brain were not sensitive to the prooxidative effects of cadmium, i.e., no increase in LPO level was observed, rather a significant decrease was noticed in the ovary tissue in the presence of the highest concentration of CdCl_2_. Notably, baseline LPO levels were higher in brain homogenates compared to the analyzed tissues.

In turn, when homogenates of the thyroid, the ovary, the liver, and the kidney were incubated in the presence of melatonin, LPO levels were generally comparable to control values. In contrast, in the case of brain homogenate, incubation with melatonin resulted in a reduction in LPO levels (Figure 1B), reaching statistical significance at the highest melatonin concentrations (5.0 and 2.5 mM). These findings indicate a tissue-specific and concentration-dependent antioxidative effect of melatonin.

**Figure 2:**
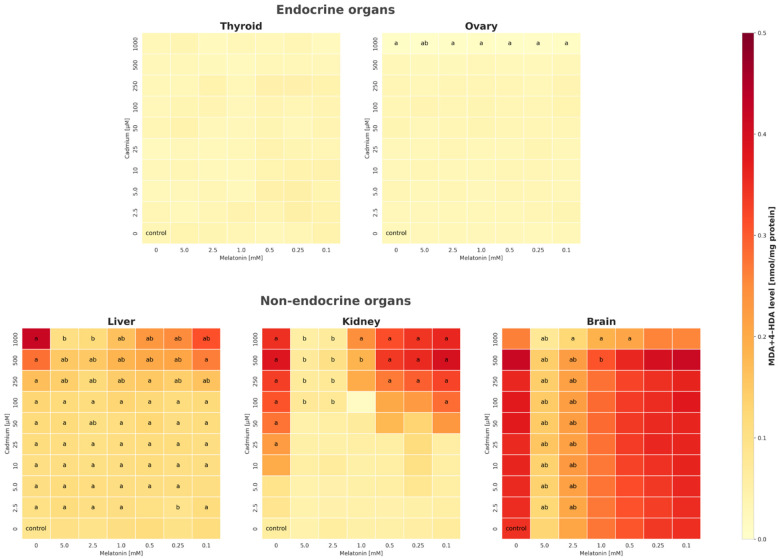
Lipid peroxidation (LPO), assessed as the level of malondialdehyde + 4-hydroxyalkenals (MDA + 4-HDA), in porcine tissue homogenates. Homogenates were incubated in the presence or absence of cadmium chloride (CdCl_2_) (0, 2.5, 5.0, 10, 25, 50, 100, 250, 500 and 1000 µM) and melatonin (0, 0.10, 0.25, 0.50, 1.0, 2.5 and 5.0 mM). LPO levels are expressed as nmol/mg protein. Data are presented as the mean. Color intensity reflects the level of LPO. “a” *p* < 0.05 vs. control within the same tissue; “b” *p* < 0.05 vs. samples treated with the same concentration of CdCl_2_ without melatonin within the same tissue.

Co-incubation of porcine tissue homogenates with CdCl_2_ and melatonin resulted in tissue-specific and concentration-dependent changes in LPO, measured as MDA + 4-HDA levels (Figure 2); for clarity, results related to melatonin alone and cadmium alone are also presented in Figure 2 and described below.

In endocrine tissues (the thyroid and the ovary), LPO levels remained relatively stable across the tested combinations of cadmium and melatonin, with no consistent Cd-induced increase or evident protective effect of melatonin, although in ovarian homogenates, Cd at 1000 µM resulted in a significant decrease in LPO levels compared to control (shown also in Figure 1A), and this effect was further enhanced in the presence of melatonin (5.0 mM).

In contrast, non-endocrine tissues exhibited more pronounced and dynamic responses. In liver and kidney homogenates, increasing cadmium concentrations were associated with elevated LPO levels, particularly at higher concentrations. In these tissues, melatonin exerted a tissue-specific protective effect against Cd-induced lipid peroxidation. In the liver homogenates, melatonin attenuated Cd-induced oxidative damage already at lower concentrations (from 0.1 mM) within the range of 250–1000 µM Cd. In contrast, in kidney homogenates, this protection was limited to higher melatonin concentrations (2.5 and 5.0 mM) and was observed for Cd concentrations in the range of 100–1000 µM.

Brain tissue showed a distinct response pattern, characterized by the highest LPO levels among all analyzed tissues. While cadmium exposure maintained elevated LPO levels, melatonin reduced lipid peroxidation across the entire range of Cd concentrations, reaching statistical significance at the highest melatonin concentrations (2.5 and 5.0 mM).

Overall, the interaction between cadmium and melatonin was tissue-dependent, with an evident protective effect of melatonin observed in non-endocrine tissues, especially in the liver.

## 3. Discussion

The present study demonstrates marked tissue-specific differences in susceptibility to cadmium-induced oxidative damage and in responsiveness to melatonin treatment.

Cadmium is well known to induce oxidative stress indirectly, mainly through excessive generation of reactive oxygen species (ROS), impairment of antioxidant defense systems, and disruption of mitochondrial function [8,9,10,11]. Mitochondria are considered one of the major intracellular sources of reactive oxygen species and are also important targets of cadmium toxicity. Cadmium has been shown to impair mitochondrial function, including disruption of the electron transport chain, which may lead to excessive ROS generation and further amplification of oxidative stress. Thus, mitochondrial dysfunction and ROS overproduction may constitute a self-perpetuating cycle contributing to cadmium-induced cellular damage [22,23]. Although mitochondrial function was not assessed in the present study, such mechanisms may have contributed to the observed lipid peroxidation in cadmium-sensitive tissues. Increased lipid peroxidation observed in liver and kidney homogenates in the present study is consistent with previous findings indicating that these organs are particularly susceptible to Cd-induced oxidative damage [24]. This vulnerability is most likely related to their important roles in metabolism, detoxification, and cadmium accumulation [25]. Indeed, the kidney and the liver are considered major target organs for cadmium toxicity due to the tendency of this metal to accumulate in these tissues during prolonged exposure [24]. Although cadmium may also accumulate in other organs, including endocrine and nervous tissues, the liver and the kidney are generally considered the primary sites of cadmium deposition and toxicity during chronic exposure [10,11]. Nevertheless, cadmium accumulation has also been demonstrated in endocrine organs, including the thyroid and the ovary, where this metal may disrupt hormonal homeostasis, impair cellular redox balance, and induce structural and functional alterations [26,27]. In the thyroid gland, cadmium exposure has been associated with disturbances in thyroid hormone metabolism and oxidative damage, whereas in the ovary, cadmium may affect steroidogenesis, follicular integrity, and reproductive function [26,27,28]. In turn, brain tissue is considered particularly susceptible to oxidative stress due to its high metabolic activity, intensive oxygen consumption, and membrane lipid composition rich in polyunsaturated fatty acids susceptible to peroxidation [29,30,31].

It is worth mentioning that tissue-dependent responses to prooxidants were also observed in our previous studies. For example, the thyroid was much less sensitive to Fenton reaction substrates compared to the ovary [32]. Similarly, after exposure to potassium iodate (KIO_3_), the thyroid exhibited lower susceptibility to lipid peroxidation compared to the spleen, ovary, and intestine [15]. Thus, the thyroid gland, as an organ of oxidative nature [21], is generally characterized by weaker susceptibility to exogenous prooxidants, and this was also observed in the current study in response to cadmium. This relative resistance may result from adaptation to continuous physiological exposure to reactive oxygen species generated during thyroid hormone biosynthesis and from efficient antioxidant defense systems required to maintain redox homeostasis. In contrast to the relatively lower susceptibility of the thyroid observed in the present and previous studies, ovarian tissue appears to be more vulnerable to cadmium-related endocrine and redox disturbances. Experimental studies have shown that cadmium may alter estrous cycle duration, estradiol concentrations, steroidogenic and folliculogenic gene expression, and signaling pathways involved in cell survival, including mTOR/ERK1/2-related signaling, endoplasmic reticulum (ER) stress, autophagy, and apoptosis [33,34]. Importantly, melatonin co-treatment was reported to prevent or attenuate many of these alterations, including ER-stress marker activation, changes in the Bax/Bcl-2 ratio, caspase-3 activation, and ovulatory dysfunction [33,34]. Thus, ovarian susceptibility to cadmium may involve not only lipid peroxidation but also broader signaling disturbances related to ER stress, apoptosis, and follicular function.

Melatonin is recognized as one of the most potent endogenous antioxidants and free radical scavengers. In addition to its direct free radical-scavenging activity, melatonin has been reported to modulate endogenous antioxidant defense systems, including antioxidant enzymes and redox-regulating pathways [14]. Its indirect antioxidant effects include stimulation of cellular antioxidant defenses, increased expression of antioxidant-related genes, enhanced levels of reduced glutathione, and increased activity of key antioxidant enzymes. Furthermore, melatonin may initiate an antioxidant cascade, independently of melatonin receptor signaling, whereby its first-, second-, and third-generation metabolites also function as free radical scavengers [14,35]. Thus, the protective effects observed in the present study may result not only from direct ROS neutralization but also from activation of endogenous antioxidant defenses and the antioxidant activity of melatonin-derived metabolites. Melatonin has also been shown to influence epigenetic mechanisms, including DNA methylation, which is followed by changes in various gene expressions [18]. It is also worth mentioning that melatonin may exert immunomodulatory effects, as shown in our recent in vitro study in which melatonin reduced surface FcγRIII/CD16-related parameters in porcine peripheral blood mononuclear cells, supporting its broader role in the regulation of immune cell phenotype [36].

In the present study, melatonin attenuated cadmium-induced lipid peroxidation in non-endocrine tissues, although the magnitude of protection differed between organs. The protective effect was observed over a broader range of experimental conditions in the liver, which is consistent with recent hepatic models showing that melatonin reduces cadmium-associated oxidative stress, lipotoxicity, inflammatory responses, and cytotoxicity, including under free fatty acid-induced metabolic stress [37,38]. However, because lipidomic and metabolic endpoints were not assessed here, these mechanisms should be considered only as a broader context for the hepatic responsiveness observed in the present model.

By contrast, kidney homogenates required higher melatonin concentrations to achieve a significant reduction in LPO levels. In vivo studies of cadmium-induced nephrotoxicity indicate that melatonin may also restore renal antioxidant status and attenuate inflammatory, fibrotic, and apoptotic responses, including changes in superoxide dismutase (SOD), glutathione (GSH), catalase (CAT), MDA, tumor necrosis factor-alpha, inducible nitric oxide synthase, transforming growth factor-beta 1, alpha-smooth muscle actin, collagen I, and caspase-3 [39]. Since these markers were not assessed in the present in vitro homogenate model, such mechanisms should be interpreted as possible downstream events relevant mainly to chronic in vivo cadmium exposure. Overall, the observed liver–kidney differences may reflect tissue-specific variations in endogenous antioxidant capacity, membrane lipid composition, cadmium-handling mechanisms, and metallothionein-mediated metal sequestration.

These differences may reflect tissue-specific variations in endogenous antioxidant capacity, membrane lipid composition, or cadmium-handling mechanisms, including metallothionein-mediated metal sequestration. Metallothioneins represent an important component of Cd toxicology and may provide a broader mechanistic context for tissue-specific Cd responses. These low-molecular-weight, cysteine-rich proteins bind cadmium through thiol groups, thereby reducing the pool of free Cd ions capable of interacting with sensitive intracellular targets. In vivo, metallothionein-bound Cd is also involved in cadmium redistribution and renal accumulation, since Cd–metallothionein complexes may be filtered in the kidney by the glomerulus and reabsorbed by proximal tubular cells. Following lysosomal degradation, Cd ions can be released and may contribute to tubular injury when local metallothionein-mediated sequestration is insufficient [40]. Although such inter-organ transport mechanisms are not directly applicable to the present in vitro tissue model, differences in basal metallothionein content, inducibility, Cd/Zn balance within metallothionein, and local cadmium-binding capacity may represent potential factors influencing tissue susceptibility to cadmium-induced oxidative damage.

Of note, brain homogenates exhibited the highest baseline LPO levels among all analyzed tissues, which most likely reflects the particularly high susceptibility of nervous tissue to oxidative processes [41]. The brain is characterized by intensive oxygen consumption, high metabolic activity, elevated content of polyunsaturated fatty acids susceptible to peroxidation, and relatively limited antioxidant defense capacity [29,30,31]. Together, these features favor enhanced generation of reactive oxygen species and high basal lipid peroxidation. In the present study, melatonin reduced the basal level of LPO (autooxidation) in brain homogenates. This finding is consistent with in vivo evidence, for example from the rat hippocampus, showing that chronic cadmium exposure increased lipid peroxidation/TBARS and nitric oxide levels, decreased SOD and CAT activities, and was associated with affective and cognitive impairments as well as neuronal loss in the CA3 region, whereas melatonin attenuated these biochemical, histological, and behavioral alterations [42]. These findings support the view that nervous tissue, because of its high basal oxidative status, lipid composition, and responsiveness to melatonin, may be particularly sensitive to the antioxidative activity of this indoleamine. This effect may additionally be favored by the ability of melatonin to easily penetrate biological membranes and accumulate in nervous tissue [43,44,45]. Nevertheless, a decrease in spontaneous autooxidation in isolated tissue homogenates should be interpreted cautiously, since physiological redox processes occur in all tissues and are not necessarily pathological.

Interestingly, cadmium-induced lipid peroxidation, as well as protective effects of melatonin, were also demonstrated in an in vivo hamster model involving the brain, the liver, and the kidney [46]. Although the experimental design differed substantially from the present in vitro study, the consistency of the findings, at least regarding the liver and the kidney, may suggest that both prooxidative effects of cadmium and antioxidative effects of melatonin result also from their direct actions without the participation of systemic circulation and systemic metabolic processes.

The limitations of the present study are as follows. The most important thing is that the study was performed under in vitro conditions using porcine tissue homogenates. Although porcine tissues are widely recognized as physiologically and biochemically similar to human organs, our observations are limited to experimental conditions that differ from those occurring in living organisms. In addition, the use of tissue homogenates rather than intact cells or whole organs does not allow evaluation of cellular integrity, intracellular signaling pathways, cadmium metabolism, or systemic regulatory mechanisms involved in oxidative stress responses.

Another limitation is that oxidative damage was evaluated only with respect to one macromolecule, i.e., membrane lipids, and only one marker of lipid peroxidation (MDA + 4-HDA) was used. Furthermore, the present study focused exclusively on melatonin as a protective antioxidative agent. Therefore, it cannot be excluded that other antioxidants may reveal different or even stronger protective effects against cadmium-induced oxidative damage. Additionally, the present study did not assess the activity of antioxidant enzymes or the involvement of redox-regulating pathways. Therefore, the mechanisms underlying cadmium-induced oxidative damage and the protective effects of melatonin could not be evaluated directly and should be investigated in future studies.

It should also be noted that each tissue homogenate represented one pooled biological sample prepared from tissues collected from 20 animals. Thus, although experiments were repeated and performed in duplicate, the obtained results should be interpreted as technical rather than fully independent biological replicates. Nevertheless, pooling of tissues reduced inter-individual variability and enabled comparative assessment of tissue-specific oxidative responses under standardized experimental conditions.

At present, the biological significance of the obtained findings requires further validation under in vivo conditions. Nonetheless, the present results demonstrate evident tissue-specific differences in susceptibility to cadmium-induced oxidative damage to membrane lipids, as well as differences in responsiveness to melatonin treatment. These observations may contribute to a better understanding of mechanisms underlying heavy metal-induced oxidative stress and antioxidant protection in different tissues.

In summary, the present findings confirm that cadmium exerts tissue-dependent prooxidative effects, with non-endocrine tissues important for metabolic processes and detoxification, i.e., the liver and the kidney, showing greater susceptibility to oxidative damage compared to endocrine organs. At the same time, melatonin demonstrated protective effects against cadmium-induced lipid peroxidation; however, the effectiveness of this protection varied depending on the tissue type and experimental conditions. The obtained results support the view that melatonin may represent a potentially beneficial protective agent against oxidative damage induced by cadmium and possibly other environmental toxicants.

## 4. Materials and Methods

### 4.1. Ethical Considerations

According to the Polish Act on the Protection of Animals Used for Scientific or Educational Purposes, dated 15 January 2015 (which implements Directive 2010/63/EU of the European Parliament and the Council of 22 September 2010, on the protection of animals used for scientific purposes), using animals solely for organ or tissue collection does not require approval from the Local Ethics Committee. Moreover, in this study, no experimental animals were used; instead, porcine tissues were obtained from animals in a slaughterhouse as part of the routine slaughter process for consumption. Additionally, a separate statement from the Ethics Committee of the Medical University of Lodz, obtained on 20 October 2021, confirmed adherence to the above directive.

### 4.2. Chemicals

All chemicals used in the study were of analytical grade and were commercially sourced. Melatonin and cadmium chloride (CdCl_2_) were purchased from Sigma (St. Louis, MO, USA). Ethanol (C_2_H_5_OH) (96%) was obtained from Stanlab (Lublin, Poland), and the LPO-568 kit for LPO was acquired from Enzo Life Science (Farmingdale, NY, USA).

### 4.3. Animals

Porcine tissues were obtained from twenty (20) pigs of both sexes at a local slaughterhouse. All procedures involving animals complied with the European Community Council Regulation (1099/2009/EC) on the protection of animals at the time of killing. The animals were sexually mature, as confirmed by their age (8–9 months) and body mass [118 ± 3.8 (SD) kg], and were assessed as healthy and free from pathological conditions by the attending veterinary officer responsible for animal health and slaughterhouse hygiene. Within 5 min following slaughter, selected tissues, including the thyroid glands, ovaries, kidneys (renal cortex), liver (left lateral lobe), and brain, were excised, immediately frozen in solid CO_2_, and stored at −80 °C until further experimental analysis.

### 4.4. Experimental Steps

Tissues from the thyroid, ovary, liver, kidney, and brain were homogenized in a 20 mM Tris-HCl buffer (pH 7.4) at a 10% (*w*/*v*) concentration, kept ice-cold, and subsequently incubated for 30 min at 37 °C with the test substances. Melatonin was dissolved in absolute ethanol, with a final ethanol concentration of 1% (*v*/*v*) in the incubation mixture. A cadmium compound was prepared using Tris-HCl buffer.

In initial tests, we evaluated whether the available cadmium compound could induce lipid peroxidation. In the main experiments, co-incubations were conducted with a single antioxidant in homogenates of the thyroid, ovary, liver, kidney or brain, as detailed below:

Experiment 1: CdCl_2_ (1000, 500, 250, 100, 50, 25, 10, 5.0 and 2.5 μM) with/without melatonin (5.0, 2.5, 1.0, 0.50, 0.25 and 0.10 mM) in the thyroid.

Experiment 2: CdCl_2_ (1000, 500, 250, 100, 50, 25, 10, 5.0 and 2.5 μM) with/without melatonin (5.0, 2.5, 1.0, 0.50, 0.25 and 0.10 mM) in the ovary.

Experiment 3: CdCl_2_ (1000, 500, 250, 100, 50, 25, 10, 5.0 and 2.5 μM) with/without melatonin (5.0, 2.5, 1.0, 0.50, 0.25 and 0.10 mM) in the liver.

Experiment 4: CdCl_2_ (1000, 500, 250, 100, 50, 25, 10, 5.0 and 2.5 μM) with/without melatonin (5.0, 2.5, 1.0, 0.50, 0.25 and 0.10 mM) in the kidney.

Experiment 5: CdCl_2_ (1000, 500, 250, 100, 50, 25, 10, 5.0 and 2.5 μM) with/without melatonin (5.0, 2.5, 1.0, 0.50, 0.25 and 0.10 mM) in the brain.

The cadmium concentrations used in the experiment represent a cross-section ranging from the highest concentrations achievable in vitro (limited by solubility) to lower concentrations that no longer produced measurable pro-oxidative effects. The concentrations of melatonin were selected based on previous experiments conducted in our laboratory [15]. These represent the highest concentrations achievable in vitro due to limited solubility and showed the most effective performance in our experimental model [15,16,32]. All experiments were conducted in duplicate and repeated three times.

Each homogenate was prepared as a pooled biological sample consisting of tissues (either the thyroid or the ovary, or the liver, or the kidney, or the brain) collected from 20 individual animals. As such, all experiments were performed on one composite biological sample per tissue, with each treatment condition tested in duplicate and repeated in three independent experimental runs to ensure technical reproducibility.

### 4.5. Measurement of Lipid Peroxidation (LPO) Products

The concentration of MDA + 4-HDA was measured as an indicator of LPO in tissue homogenates. The LPO-586 kit was applied as a protocol in the whole described procedure. In brief, homogenates were centrifuged at 3000× *g* for 10 min at 4 °C. After collecting the supernatant, each experiment was performed in duplicate. A 200 μL sample of the supernatant was combined with 650 μL of a methanol solution (1:3, *v*/*v*) containing the chromogenic agent N-methyl-2-phenylindole, then vortexed. Subsequently, 150 μL of methanesulfonic acid (15.4 M) was added, and the mixture was incubated at 45 °C for 40 min. The reaction between MDA + 4-HDA and N-methyl-2-phenylindole forms a chromophore, which was measured spectrophotometrically at 586 nm using a 10 mM 4-hydroxynonenal (4-HNE) solution as a standard. Lipid peroxidation levels were expressed as the amount of MDA + 4-HDA (nmol) per mg of protein. Protein content was determined using the Bradford method [47], with bovine serum albumin as the standard.

### 4.6. Statistical Analyses

The data were analyzed statistically using a one-way analysis of variance (ANOVA), followed by the Student–Newman–Keuls test, for cases involving more than two experimental groups (such as different concentrations of cadmium alone or cadmium combined with various concentrations of melatonin). For comparisons between two independent groups (cadmium alone versus cadmium plus melatonin), an unpaired Student’s *t*-test was applied. A level of *p* < 0.05 was considered statistically significant.

## 5. Conclusions

This study demonstrates that cadmium-induced oxidative damage is highly tissue-specific, with the liver and the kidney exhibiting the highest sensitivity to lipid peroxidation. This vulnerability is likely due to the specificity of these organs, including their high metabolic activity, intensive oxygen consumption, and their role as primary sites of cadmium accumulation and detoxification, involving, for example, the synthesis of metal-binding proteins such as metallothionein. Melatonin serves as an effective antioxidant against cadmium toxicity, though its protective efficacy varies significantly across different organs. The liver exhibited the most consistent response to melatonin across a wide range of concentrations, potentially due to its high metabolic turnover and the hormone’s synergy with hepatic antioxidant enzymes. In contrast, the kidney required significantly higher doses of melatonin to achieve protective effects. Of note, brain tissue demonstrated particularly high baseline lipid peroxidation levels, suggesting its high susceptibility to oxidative stress. Interestingly, the thyroid’s resistance to cadmium-induced LPO suggests that its high physiological oxidative activity (indispensable for hormone synthesis) paradoxically makes the organ less vulnerable to further pro-oxidative factors. Overall, these findings suggest that cadmium toxicity occurs via, among others, a mechanism of lipid peroxidation. Additionally, they support the potential of melatonin as a preventive agent against cadmium-induced oxidative damage, similarly to what has been observed for other heavy metals.

## Data Availability

The datasets used and/or analyzed during the current study are available from the corresponding author on reasonable request.
